# Efficacy of Mirikizumab in Patients with Prior Ustekinumab Exposure: A Case Series

**DOI:** 10.1093/ibd/izaf018

**Published:** 2025-04-08

**Authors:** Tsunaki Sawada, Masanao Nakamura, Takeshi Yamamura, Keiko Maeda, Eri Ishikawa, Kentaro Murate, Hiroki Kawashima

**Affiliations:** Department of Endoscopy, Nagoya University Hospital, 65 Tsurumai-cho, Showa-ku, Nagoya, Japan; Department of Endoscopy, Nagoya University Hospital, 65 Tsurumai-cho, Showa-ku, Nagoya, Japan; Department of Gastroenterology and Hepatology, Nagoya University Graduate School of Medicine, 65 Tsurumai-cho, Showa-ku, Nagoya, Japan; Department of Gastroenterology and Hepatology, Nagoya University Graduate School of Medicine, 65 Tsurumai-cho, Showa-ku, Nagoya, Japan; Department of Gastroenterology and Hepatology, Nagoya University Graduate School of Medicine, 65 Tsurumai-cho, Showa-ku, Nagoya, Japan; Department of Gastroenterology and Hepatology, Nagoya University Graduate School of Medicine, 65 Tsurumai-cho, Showa-ku, Nagoya, Japan; Department of Gastroenterology and Hepatology, Nagoya University Graduate School of Medicine, 65 Tsurumai-cho, Showa-ku, Nagoya, Japan

## Case Series

Interleukin-23 (IL-23) consists of 2 subunits: p40, shared with interleukin-12 (IL-12), and the unique p19 subunit.^1^ Mirikizumab, a humanized IgG4-variant monoclonal antibody that binds to p19, has shown efficacy in treating ulcerative colitis (UC).^2^ However, patients previously treated with ustekinumab, a monoclonal antibody targeting p40, were excluded from phase 3 trials of mirikizumab, resulting in a lack of clinical evidence for such cases.

This case series included 10 patients with clinically active UC who had prior ustekinumab exposure and were initiated on mirikizumab therapy at the Nagoya University Hospital (Nagoya, Japan) between July 2023 and June 2024. The cohort included 8 male patients with a median age of 39 years (range: 23–67) (Table 1). The median disease duration was 4 years (range: 1–27). One patient was a primary nonresponder to ustekinumab, whereas the remaining 9 were secondary nonresponders. All patients received subcutaneous ustekinumab at a dose of 90 mg every 8 weeks as maintenance therapy, with no 6-week dosing or re-induction performed. Eight patients had prior exposure to anti-TNF agents, 7 to vedolizumab, and 4 to JAK inhibitors. In one case, IM was used concomitantly. Three patients presented with a Mayo endoscopic sub-score of 3, and a median Lichtiger Clinical Activity Index (CAI) of 8 (range: 5-10).

**Table 1.** Baseline characteristics and outcomes at 3 months.

Case	Sex	Age (yr)	Disease duration (yr)	BMI	Disease type	Response to UST	Prior Exposure to Biologics and JAKi	IM Use	CAI	MES	Alb (g/dL)	CRP (mg/L)	Outcome
1	Male	67	27	23.2	Left-sided	SNR	Anti-TNF (+), VDZ (+), JAKi (−)	−	5	2	3.7	2.3	Clinical remission
2	Male	33	4	22.6	Pan-colitis	SNR	Anti-TNF (−), VDZ (+), JAKi (−)	−	7	2	3.3	0.2	Clinical remission
3	Male	23	4	16.9	Pan-colitis	SNR	Anti-TNF (+), VDZ (+), JAKi (+)	−	8	3	4.2	7.8	Clinical remission
4	Female	35	6	25	Pan-colitis	SNR	Anti-TNF (+), VDZ (−), JAKi (+)	−	8	NA	3.9	4.2	Clinical remission
5	Male	42	3	17.4	Pan-colitis	SNR	Anti-TNF (+), VDZ (+), JAKi (−)	−	5	2	3.8	0.1	Nonresponder
6	Male	50	12	20.2	Pan-colitis	SNR	Anti-TNF (+), VDZ (+), JAKi (−)	−	8	2	3.9	7.1	Clinical remission
7	Male	53	1	20.2	Pan-colitis	SNR	Anti-TNF (−), VDZ (−), JAKi (−)	−	10	3	3.6	0.4	Clinical remission
8	Male	27	2	32.7	Left-sided	PNR	Anti-TNF (+), VDZ (−), JAKi (+)	−	8	3	4.4	2.4	Partial responder
9	Male	40	12	19	Pan-colitis	SNR	Anti-TNF (+), VDZ (+), JAKi (+)	−	6	NA	3.9	3.7	Nonresponder
10	Female	38	1	33.5	Pan-colitis	SNR	Anti-TNF (+), VDZ (+), J AKi (−)	+	8	2	3.8	2.2	Clinical remission

Abbreviations: Alb, serum albumin level; anti-TNF, anti-TNF alpha agents; BMI, body mass index; CAI, Lichtiger Clinical Activity Index; CRP, C-reactive protein; IM, immunomodulator; JAKi, Janus kinase inhibitors; MES, Mayo Endoscopic Subscore; NA, not available; PNR, primary no responder; SNR, secondary no responder; UST, ustekinumab; VDZ, vedolizumab.

At 3 months after initiating mirikizumab, 7 patients achieved corticosteroid-free clinical remission, defined as a CAI ≤ 3 without corticosteroid use. One patient achieved a clinical response, defined as a reduction in CAI ≥ 3 without remission, while 2 patients discontinued treatment due to primary nonresponse. Among those achieving clinical remission, 5 did so within 1 month, 1 within 2 months, and 1 within 3 months. In the 8 patients excluding the 2 primary nonresponders, CAI decreased from 8 (5-10) to 2 (1-8) at 1 month, 1.5 (0-4) at 2 months, and 1.5 (0-4) at 3 months. Similarly, C-reactive protein levels improved from 2.35 mg/L (range: 0.2-7.8) to 1.55 mg/L (range: 0.1-5.4), and serum albumin levels changed from a median of 3.85 g/dL (range: 3.3-4.4) to 3.9 g/dL (range: 3.3-4.7) at 3 months. No adverse events were observed.

## Discussion

To the best of our knowledge, this is the first case series reporting the efficacy of mirikizumab in patients with prior ustekinumab failure. Although this study included only moderately active cases, corticosteroid-free remission was achieved in 7 of 10 cases. The efficacy of mirikizumab may be attributed to its higher binding affinity for IL-23 compared to ustekinumab.^3^ However, several factors may have influenced the outcomes. For example, only one patient was a primary nonresponder to ustekinumab, and cytokine dynamics may have been influenced by medications administered between ustekinumab and mirikizumab. Large-scale studies are required to identify predictors of mirikizumab efficacy in patients treated with ustekinumab.
